# Progression criteria in trials with an internal pilot: an audit of publicly funded randomised controlled trials

**DOI:** 10.1186/s13063-019-3578-y

**Published:** 2019-08-09

**Authors:** Esther Herbert, Steven A. Julious, Steve Goodacre

**Affiliations:** School of Health and Related Research, University of Sheffield, Regent Court, Regent Street, Sheffield, S1 4DA UK

**Keywords:** Internal pilot, Audit, Feasibility, Recruitment

## Abstract

**Background:**

With millions of pounds spent annually on medical research in the UK, it is important that studies are spending funds wisely. Internal pilots offer the chance to stop a trial early if it becomes apparent that the study will not be able to recruit enough patients to show whether an intervention is clinically effective. This study aims to assess the use of internal pilots in individually randomised controlled trials funded by the Health Technology Assessment (HTA) programme and to summarise the progression criteria chosen in these trials.

**Methods:**

Studies were identified from reports of the HTA committees’ funding decisions from 2012 to 2016. In total, 242 trials were identified of which 134 were eligible to be included in the audit. Protocols for the eligible studies were located on the NIHR Journals website, and if protocols were not available online then study managers were contacted to provide information.

**Results:**

Over two-thirds (72.4%) of studies said in their protocol that they would include an internal pilot phase for their study and 37.8% of studies without an internal pilot had done an external pilot study to assess the feasibility of the full study. A typical study with an internal pilot has a target sample size of 510 over 24 months and aims to recruit one-fifth of their total target sample size within the first one-third of their recruitment time.

There has been an increase in studies adopting a three-tiered structure for their progression rules in recent years, with 61.5% (16/26) of studies using the system in 2016 compared to just 11.8% (2/17) in 2015. There was also a rise in the number of studies giving a target recruitment rate in their progression criteria: 42.3% (11/26) in 2016 compared to 35.3% (6/17) in 2015.

**Conclusions:**

Progression criteria for an internal pilot are usually well specified but targets vary widely. For the actual criteria, red/amber/green systems have increased in popularity in recent years. Trials should justify the targets they have set, especially where targets are low.

## Background

In the financial year 2015/16, the National Institute for Health Research (NIHR) spent £247.9 million on their research programmes and almost a third of this was spent on their Health Technology Assessment (HTA) programme [1]. With such large amounts of public money being spent on health research, it is important that the funds are used wisely and that money is not wasted on trials which are not likely to succeed.

An internal pilot is a phase in a trial after which progress is assessed against pre-specified targets/criteria [2, 3]. They are an opportunity to stop trials which are not likely to reach their recruitment, retention or site set-up targets (among others). Unlike an external pilot, data collected during the internal pilot phase contribute towards the final analyses of a trial. This makes internal pilots potentially more cost-effective than running an external pilot followed by a full trial. Including an internal pilot in a study allows funders to take on more risky trials, such as trials where recruitment to time and target is uncertain due to a lack of previous research in the clinical area or a trial population with a rare disease; if the internal pilot shows that the trial is not feasible, it can be stopped short to save resources.

Internal pilots give trialists the opportunity to investigate other elements of the trial such as a sample size re-estimation [4], assessments of futility and adherence to intervention. However, for the purpose of this research the focus is on internal pilots as a means to evaluate or monitor study recruitment, and other progression criteria specified by audited trials were ignored.

Trials fail for a variety of reasons including: not recruiting the target sample size; higher levels of drop-out or non-compliance than planned for; or flaws in the trial design such as an impractical method of randomisation. Recruitment is a key area of interest since failing to hit a recruitment target could leave a trial with less power to detect a clinically meaningful and statistically significant result. According to an audit of randomised controlled trials (RCTs) funded by the NIHR HTA programme, only 56% (85/151) of studies achieved their target sample size [5].

This article aims to provide a summary of the continuation criteria used in trials with internal pilots funded by the NIHR HTA programme as well specific examples of good progression criteria.

## Methods

### Identifying trials

Funding outcomes from the HTA’s Clinical Evaluation and Trials (CET) and Commissioning Committees [6] were reviewed for meetings between February 2012 and November 2016 inclusive. These committees meet regularly to discuss both researcher-led proposals and responses to the HTA’s commissioned calls. The committees then make recommendations to the HTA Prioritisation Group. Trials funded by the HTA programme were chosen because it is the largest research programme within the NIHR; HTA funding made up 30% of all research programme funding in 2015/16 [1]. HTA-funded trials were also chosen because of the level of quality planning required for funding.

Funded trials were then identified on the NIHR Journals website [7], where details of funded projects are given and study protocols are uploaded. The protocols listed were used to determine whether the study was suitable for the audit. Studies were included in the audit if they were individual randomised controlled trials with a listed protocol.

### Analyses

Trials were categorised by whether they had an internal pilot, external pilot, neither or both and the proportions of trials in each were compared by year of trial funding approval. The year of funding approval was chosen instead of the year of trial start because it was believed to form a more accurate representation of the trends in pilot inclusion. Trials do not start immediately after funding approval and the period between funding decision and starting can vary a lot; this means that the start date for a trial may not be a good indicator of trends in trials practice since the protocol trial design will have been decided a varying time before.

In the paper by Avery et al. [8], recommendations were made for developing progression criteria for internal pilots. The trials in our audit were analysed to see whether they followed two of the top tips from the article:

**Table:** 

Question	Recommendation
• Were the criteria given as a stop/go decision (e.g. continuation based on meeting a set target) or as a more complex red/amber/green decision, where studies falling into the amber section would require more discussion as to whether they would continue?	• Avery et al. [8] recommended using the red/amber/green system.
• Were criteria based on recruitment targets given as a target number of patients recruited or as a target recruitment rate?	• Rates per centre per unit time were recommended since they can then be used to extrapolate the predicted full recruitment length and are not as susceptible to sites opening late.

**Fig. 1:**
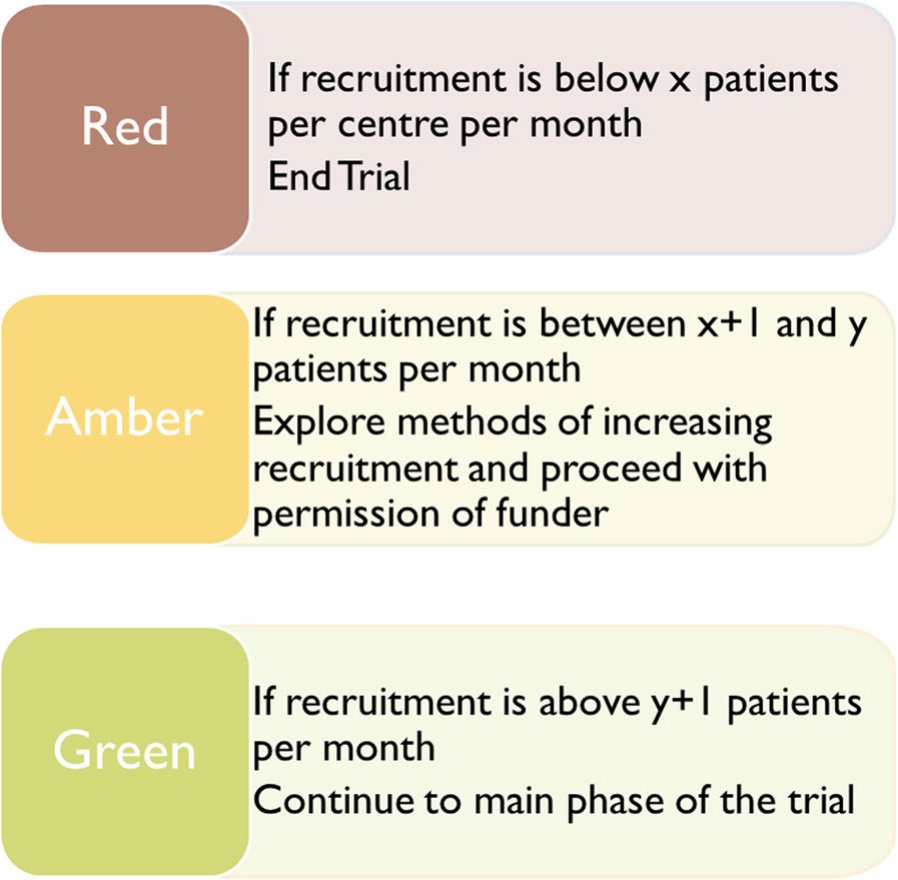
Illustration of the red/amber/green system of criteria

The red/amber/green system for progression criteria gives a three-tiered approach as illustrated in Fig. 1.

For example, the green criteria might be that trials will be allowed to continue if they recruit at 100% of their pre-specified target for that period; the amber criteria could be that if the trial recruits at less than 100% of their target but better than 60%, then things will be looked into; and the red criteria would then be that if recruitment fell to less than 60% of the target the trial would end.

Results from these questions were also compared by year of funding approval to see whether there has been a change in the types of criteria used across the years.

A further question we wished to investigate was the duration of internal pilots as a proportion of the planned full trial. In order to assess this, we looked at the length of the pilot phase in terms of months of recruitment and recruitment target. Recruitment targets for internal pilots were not always given, for example when progression targets were given as recruitment rates. In these cases, the target number of patients recruited for the pilot was extrapolated where possible.

All analyses were performed in R (version 3.5.1) [9].

## Results

Through documentation of funding decisions found on the NIHR webpage [6], 242 studies were identified. Of these, 134 (55.4%) were included in the audit. Reasons for exclusion included the following:

- The study was an external pilot/feasibility study.
- The protocol was missing.
- The study was a systematic review.

**Fig. 2:**
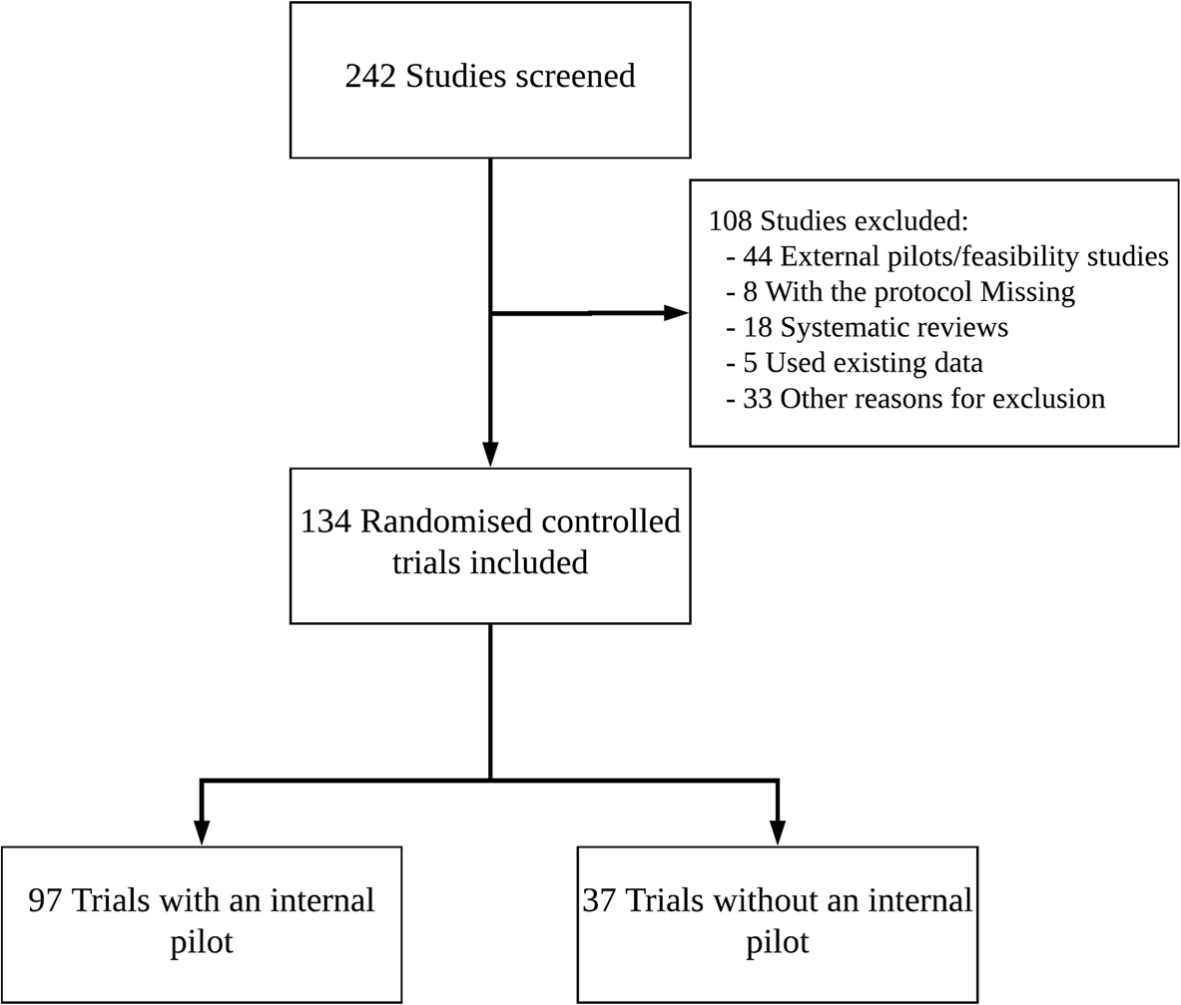
Flow of studies through the audit

Efforts were made to contact study managers for studies where the protocol was missing from the NIHR Journals library, but it was not always possible to obtain the information needed. The CONSORT-style diagram in Fig. 2 shows the flow of studies through the audit, including reasons for exclusion from the study.

On average, 26.8 (12.19) trials were approved each year and met our inclusion criteria. However, only funding decisions made in February were available for 2012. This means it is only sensible to look at data from 2013 to 2016 when assessing trends across the years but the data from 2012 have been included for completeness. For all other years, data were available for at least 3 months, with data for 1 month more available in 2013 which explains the slight increase in studies from that year.

Out of the 134 studies included in the audit, 72.4% (97/134) said that they would include an internal pilot in their protocol. Of those that did not include an internal pilot, 37.8% (14/37) had done an external pilot prior to their study approval. This means that 82.8% (111/134) of studies audited included some form of pilot/feasibility work.

The Big CACTUS study [10] approved in 2013 included an internal pilot phase having already completed an external pilot [11]. This was due to significant changes made after the external pilot, including the addition of another arm to the trial.

**Table 1:** Characteristics of the trials audited (*n* = 134) stratified by the presence of an internal pilot

		Internal pilot	No internal pilot	Total
Disease area	Cancer	14 (82.4%)	3 (17.6%)	17
	Circulatory system	6 (60.0%)	4 (40.0%)	10
	Digestive system	1 (50.0%)	1 (50.0%)	2
	Ear, nose and throat	1 (50.0%)	1 (50.0%)	2
	Eye diseases	3 (75.0%)	1 (25.0%)	4
	Infections and infestations	5 (100.0%)	0 (0.0%)	5
	Injury, occupational diseases, poisoning	5 (71.4%)	2 (28.6%)	7
	Mental and behavioural disorders	14 (60.9%)	9 (39.1%)	23
	Musculoskeletal diseases	11 (84.6%)	2 (15.4%)	13
	Neonatal diseases	2 (100.0%)	0 (0.0%)	2
	Nervous system diseases	6 (66.7%)	3 (33.3%)	9
	Pregnancy and childbirth	9 (60.0%)	6 (40.0%)	15
	Respiratory	4 (80.0%)	1 (20.0%)	5
	Skin and connective tissue diseases	3 (75.0%)	1 (25.0%)	4
	Urological and genital diseases	6 (66.7%)	3 (33.3%)	9
	Other^a^	7 (100.0%)	0 (0.0%)	7
Power	80%	12 (66.7%)	6 (33.3%)	18
	85%	1 (100.0%)	0 (0.0%)	1
	90%	75 (73.5%)	27 (26.5%)	102
	95%	1 (50.0%)	1 (50.0%)	2
	Other	8 (72.7%)	3 (27.3%)	11
Year of funding decision	2012	2 (28.6%)	5 (71.4%)	7
	2013	29 (72.5%)	11 (27.5%)	40
	2014	20 (76.9%)	6 (23.1%)	26
	2015	20 (64.5%)	11 (35.5%)	31
	2016	26 (86.7%)	4 (13.3%)	30

^a^Genetic diseases; nutritional, metabolic, endocrine; oral health; signs and symptoms; surgery; and not applicable

Table 1 presents the properties of the studies included in the audit, broken down by whether an internal pilot was included. There appears to be no difference between the types of studies with or without an internal pilot.

**Fig. 3:**
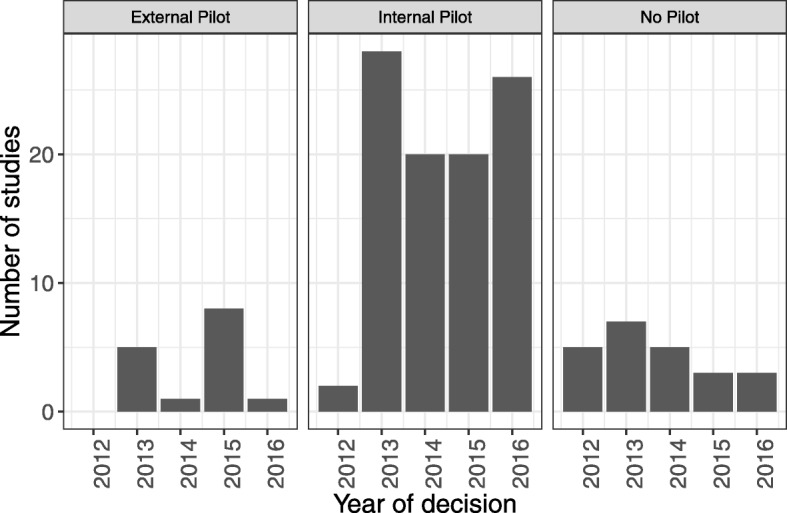
Number of studies with an internal pilot, an external pilot or no pilot, broken down by year

Figure 3 shows the breakdown of how many studies included pilots (either internal or external) by year of funding approval. The proportion of studies including an internal pilot has increased over the years but 10.0% (3/30) of RCTs approved in 2016 still did not have any pilot work either internally or externally.

Of the 97 studies that indicated in their protocol that they would include an internal pilot, 89.7% (87/97) gave criteria for progression from the pilot phase to the full trial. Table 2 presents the proportions of studies using stop/go or red/amber/green systems and whether the number or the rate was given for targets involving recruitment. The most popular format for criteria was a stop/go system with a recruitment target given in terms of the number of patients to be recruited; this was seen in 44.8% (39/87) of the studies that gave criteria.

**Table 2:** Criteria specifications for all 97 studies with an internal pilot included in the audit

Number/rate	Type of criteria		
	Red/amber/green	Stop/go	Missing
Number	18 (18.6%)	39 (40.2%)	0 (0.0%)
Number and rate	1 (1.0%)	4 (4.1%)	0 (0.0%)
Rate	10 (10.3%)	15 (15.5%)	0 (0.0%)
Missing	0 (0.0%)	0 (0.0%)	10 (10.3%)

**Fig. 4:**
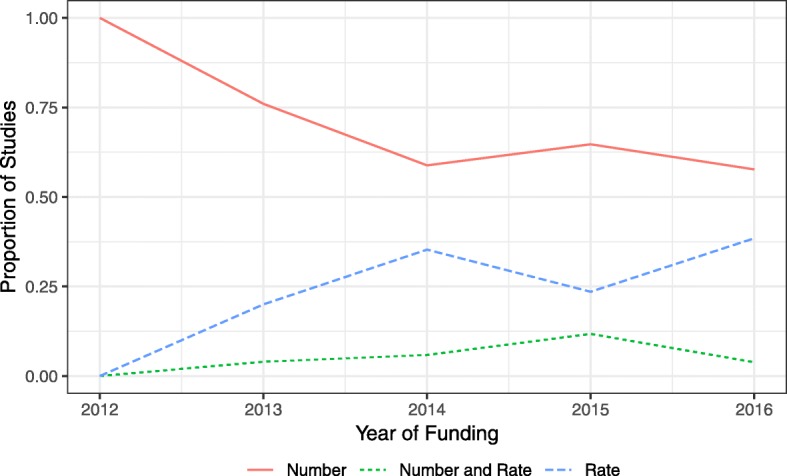
Trend in whether the recruitment rate or the number recruited was used in criteria for internal pilots

**Fig. 5:**
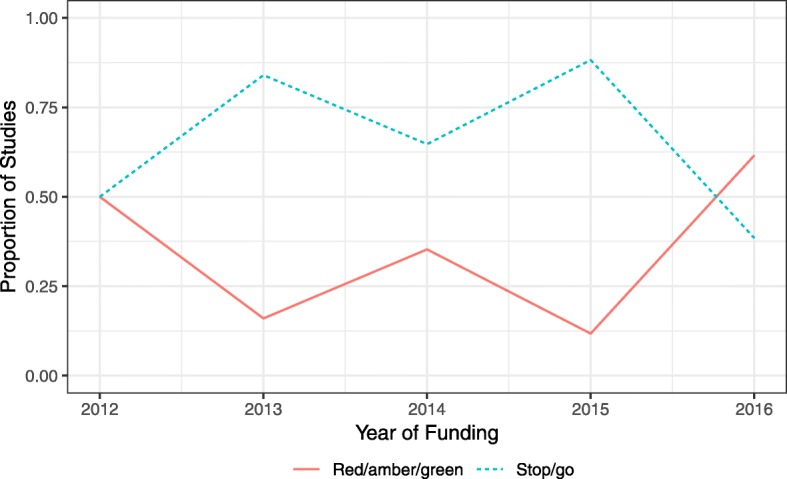
Trend in whether a red/amber/green system or a stop/go system was used for progression criteria

In 2016 there was an increase in studies adopting the red/amber/green structure for their progression rules, with 61.5% (16/26) of studies using the system compared to just 11.8% (2/17) in 2015. There was also a rise in the number of studies giving a target recruitment rate in their progression criteria: 42.3% (11/26) in 2016 compared to 35.3% (6/17) in 2015. Figures 4 and 5 show the change in the proportion of studies using different types of criteria across the years. Proportions were calculated excluding trials with an internal pilot but no progression criteria stated in the trial protocol.

Table 3 presents details of what proportion of a trial is used for the internal pilot phase. The average proportion of recruitment months used in the pilot is 33.5% (SD 12.6%). However, the average proportion of the sample size aimed to be recruited in the internal pilot was 18.5% (SD 10.4%).

**Table 3:** Recruitment properties of the studies audited, stratified by presence of an internal pilot

		Internal pilot (*N* = 97)	No internal pilot (*N* = 37)	Total (*N* = 134)
Total target sample size	*n*	97	36	133
	Mean (SD)	945.1 (1400.0)	1171.8 (1460.3)	1006.5 (1414.6)
	Median (IQR)	510.0 (350.0, 900.0)	625.0 (395.0, 1,349.2)	533.0 (360.0, 1,044.0)
	Minimum, maximum	120, 9920	100, 8000	100, 9920
Recruitment target for internal pilot	*n*	69	–	69
	Mean (SD)	135.2 (156.8)	–	135.2 (156.8)
	Median (IQR)	100.0 (48.0, 162.0)	–	100.0 (48.0, 162.0)
	Minimum, maximum	20, 1165	–	20, 1165
Proportion of sample size aimed to be recruited in internal pilot (%)	*n*	69	–	69
	Mean (SD)	18.5 (10.4)	–	18.5 (10.4)
	Median (IQR)	15.8 (10.2, 25.0)	–	15.8 (10.2, 25.0)
	Minimum, maximum	2.3, 50	–	2.3, 50
Length of recruitment for full study (months)	*n*	96	35	131
	Mean (SD)	28.6 (10.9)	22.3 (9.1)	26.9 (10.8)
	Median (IQR)	24.0 (20.8, 36.0)	20.0 (16.5, 30.0)	24.0 (18.0, 36.0)
	Minimum, maximum	11, 60	5, 45	5, 60
Length of recruitment for internal pilot (months)	*n*	96	–	96
	Mean (SD)	9.4 (5.1)	–	9.4 (5.1)
	Median (IQR)	8.5 (6.0, 12.0)	–	8.5 (6.0, 12.0)
	Minimum, maximum	3, 30	–	3, 30
Proportion of recruitment length used in internal pilot (%)	*n*	95	–	95
	Mean (SD)	33.5 (12.6)	–	33.5 (12.6)
	Median (IQR)	33.3 (25.0, 40.8)	–	33.3 (25.0, 40.8)
	Minimum, maximum	9.4, 68.8	–	9.4, 68.8
Number of centres involved in full study	*n*	91	30	121
	Mean (SD)	20.9 (22.1)	19.9 (17.1)	20.6 (20.9)
	Median (IQR)	14.0 (7.5, 25.0)	15.5 (6.0, 29.0)	14.0 (7.0, 26.0)
	Minimum, maximum	1, 120	3, 70	1, 120
Number of centres involved in internal pilot	*n*	88	–	88
	Mean (SD)	9.4 (11.6)	–	9.4 (11.6)
	Median (IQR)	6.0 (4.0, 12.0)	–	6.0 (4.0, 12.0)
	Minimum, maximum	1, 100	–	1, 100
Proportion of centres used in internal pilot (%)	*n*	86	–	86
	Mean (SD)	56.8 (31.3)	–	56.8 (31.3)
	Median (IQR)	49.0 (33.3, 100.0)	–	49.0 (33.3, 100.0)
	Minimum, maximum	9.5, 100	–	9.5, 100

*IQR* interquartile range, *SD* standard deviation

A typical study with an internal pilot having a target sample size of 510 over 24 months aims to recruit one-fifth of their total target sample size within the first one-third of their recruitment time; this ratio of proportion of sample size to proportion of recruitment length (3:5) allows for slow initial recruitment during set-up of centres. We are not looking here at a within-site lag to recruitment (i.e. a slow start caused by staff familiarising themselves with the protocol) but, rather, a lag caused by the process of setting up multiple centres whilst recruitment is ongoing. In this sense, we would expect larger multi-centre studies to have a longer lag phase across the whole study resulting in a lower, more generous ratio of the proportion of the sample size to proportion of recruitment length and we would expect a more ambitious ratio, closer to 1, for studies with only a few sites which could all be set-up and recruiting close to the start of recruitment.

**Fig. 6:**
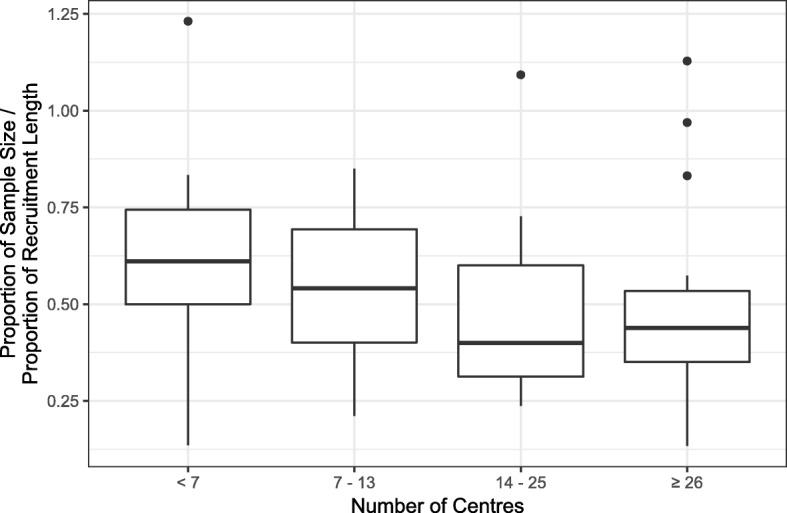
Boxplots showing the ratio of pilot recruitment target to internal pilot length stratified by the quartiles of the total number of centres in the trials

However, there appears to be little association between this ratio and the total number of centres involved in a study. Figure 6 suggests that studies with few centres (< 7) have a fractionally more ambitious ratio than studies with more centres but there is no clear association. This suggests that studies with fewer centres are not being ambitious enough with their recruitment target for their internal pilot, although most studies do not aim to open all of their sites during the internal pilot phase and we cannot exclude the possibility that overall site set-up has been allowed for in the recruitment target.

However, the target proportion of patients recruited for the internal pilot did vary depending on the relative length of the internal pilot compared to the full trial.

**Fig. 7:**
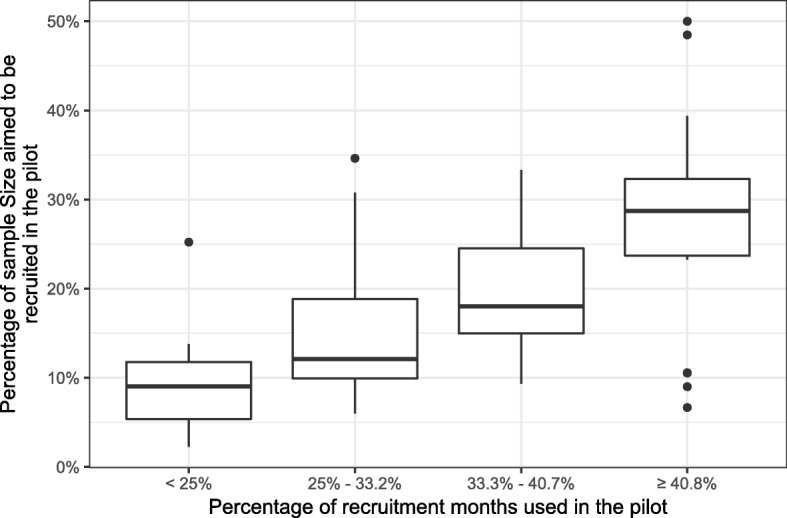
Boxplots showing the proportion of patients aimed to be recruited stratified by the proportion of the trial taken up by the internal pilot

Figure 7 shows that, as expected, the larger the proportion of the recruitment period included in the pilot phase, the larger the target sample size for the internal pilot as a proportion of the main target sample size. For example, studies whose internal pilot took up less than 25% of their recruitment months aimed to recruit 9.8% of their target sample size in this time, whereas studies whose internal pilot took up between 33.3% and 41% of their recruitment months aimed to recruit 15.3% of their target sample size.

## Exemplars

Coming up with progression criteria can often feel like an abstract concept and pre-specifying desired recruitment rates along with thresholds at which changes should be investigated or the trial should be stopped is difficult. It is helpful to look at examples to get a picture of what clear criteria look like. The following trials have given well thought out progression criteria with a red/amber/green structure and criteria based on rates.

### EASI-SWITCH trial [12]

#### Criteria

Recruitment rate (the expected recruitment rate is 1.7 patients per site per month with a 50% reduction for the first three months of site opening):

Progression without major modification if at least 75% of target reached, with analysis and resolution of any identified barriers to successful recruitment.

Progression with addition of further trial sites if between 50 and 75% of target reached.

Progression unlikely if less than 50% of target reached—this equates to, on average, 4 patients per site over the 12 month pilot period. This would be subject to detailed review of project viability by the Trial Steering Committee and HTA team. ([12], p. 23)

This criteria specification clearly states their three-tiered system which gives next steps should the trial fall into each category and allows for slower recruitment as trials open. The only potential problem is that, with the “green” target set at 75%, the trial could continue without modification towards an underpowered total sample. A “green” target of 100%, with proportionate responses to recruitment in the 75–10%% range, would address this concern.

### Prepare for Kidney Care trial [13]

#### Criteria

This trial presented their progression criteria in a helpful table (see Table 4). The protocol for the Prepare for Kidney Care trial says that if all green targets are achieved then the full trial would most likely go ahead, whereas predominantly red targets would probably illustrate that the full trial would not be feasible. The simple table clearly displays all progression criteria. In particular, the criteria for recruitment are given as rates to allow assessment of whether it is the rate or site set-up or the recruitment rate per site that is failing to meet the target. Again, it would need to be clarified that an overall rate between 85 and 100% would involve some sort of remedial action to protect against an under-powered trial.

**Table 4:** Progression criteria table from the Prepare for Kidney Care trial protocol ([13], p. 54)

	% of rate proposed	Number of sites recruiting, based on the target of 16 sites	Recruitment rate per active site per month
Green	≥ 85	14 sites or more	1.3 patients/month or more
Amber	60–84	10–13 sites	0.9–1.2 patients/month
Red	< 60	9 sites or fewer	0.8 patients/month or fewer

Adapted from [13]

## Discussion

Although they are the gold standard, clinical trials are an expensive way of making an assessment for a new health technology. To ensure optimal value for the research funding—particularly for public funding—it is good practice to have some formal decision criteria within a trial to help the trialists and the funding body decide whether it is feasible to continue with the trial.

Stop/go criteria within the trial are a method to help to determine whether the trial is feasible for the budget set where the criteria are set before the commencement of the trial and are agreed with the funder. In recent years, a traffic light system has been proposed where decisions are set out such that red equates to stop, green to go and amber to further action. This format of setting out progression criteria has increased in popularity amongst HTA-funded trials in the past few years and has the advantage of providing an amber zone that can be used to prompt remedial action rather than close down.

Choice of timing and targets for an internal pilot are important. Regardless of the timing, specifying the decision criteria as an average recruitment rate per month per site allows for a check as to whether the pilot has shown that the trial is feasible. If you multiply the recruitment rate per month per site achieved in the pilot by the number of sites and months across the whole trial you should get the total sample size.

Our investigation has looked only at studies funded by the NIHR HTA programme, as such this work has limited generalisability but will hopefully be of use to those preparing grant applications for this and other publicly funded programmes where funding is limited and there is pressure to obtain results from resources used for research. It would be interesting for further work to explore whether similar trends in internal pilots have been seen across trials funded by other streams of the NIHR and other funding bodies.

A limitation of this work is that results presented cannot, in general, be applied to cluster RCTs because they were excluded from the audit due to complexities surrounding recruitment strategies. For example, in some cluster RCTs individuals are not directly recruited; in the PLEASANT trial [14], GP practices were recruited and randomised and routine data were collected through the Clinical Practice Research Datalink (CPRD). Further work to investigate progression criteria for internal pilots within a cluster RCT would have to consider the different models of recruitment used.

## Conclusions

Progression criteria for an internal pilot are usually specified but targets vary widely. Red/amber/green systems have become more popular in recent years for specifying targets for progression. If these criteria are used with a target for the average rate per site per months in the pilot phase, this should produce the total sample size when extrapolated across the sites and duration of the full trial.

## Data Availability

The datasets used and/or analysed during the current study are available from the corresponding author on reasonable request.

## Abbreviations

CET: Clinical Evaluation and Trials
HTA: Health Technology Assessment
NIHR: National Institute for Health Research
RCT: Randomised controlled trial
SD: Standard deviation
